# Effectiveness of eHealth and mHealth Interventions Supporting Children and Young People Living With Juvenile Idiopathic Arthritis: Systematic Review and Meta-analysis

**DOI:** 10.2196/30457

**Published:** 2022-02-02

**Authors:** Sonia Butler, Dean Sculley, Derek Santos, Antoni Fellas, Xavier Gironès, Davinder Singh-Grewal, Andrea Coda

**Affiliations:** 1 School of Bioscience and Pharmacy College of Health, Medicine and Wellbeing University of Newcastle Ourimbah Australia; 2 School of Health Sciences Queen Margaret University Edinburgh United Kingdom; 3 School of Health Sciences College of Health, Medicine and Wellbeing University of Newcastle Callaghan Australia; 4 University of Vic-Central University of Catalonia Manresa Spain; 5 Department of Rheumatology Sydney Children's Hospitals Network, Randwick and Westmead Sydney Australia; 6 Department of Rheumatology John Hunter Children’s Hospital Newcastle Australia; 7 School of Women’s and Children’s Health University of New South Wales Sydney Australia; 8 Discipline of Child and Adolescent Health University of Sydney Sydney Australia; 9 Priority Research Centre Health Behaviour Hunter Medical Research Institute Newcastle Australia

**Keywords:** eHealth, mobile health, mHealth, juvenile idiopathic arthritis, pediatric, effectiveness, pain, physical activity, health-related quality of life, self-management, education, mobile phone

## Abstract

**Background:**

Juvenile idiopathic arthritis (JIA) management aims to promote remission through timely, individualized, well-coordinated interdisciplinary care using a range of pharmacological, physical, psychological, and educational interventions. However, achieving this goal is workforce-intensive. Harnessing the burgeoning eHealth and mobile health (mHealth) interventions could be a resource-efficient way of supplementing JIA management.

**Objective:**

This systematic review aims to identify the eHealth and mHealth interventions that have been proven to be effective in supporting health outcomes for children and young people (aged 1-18 years) living with JIA.

**Methods:**

We systematically searched 15 databases (2018-2021). Studies were eligible if they considered children and young people (aged 1-18 years) diagnosed with JIA, an eHealth or mHealth intervention, any comparator, and health outcomes related to the used interventions. Independently, 2 reviewers screened the studies for inclusion and appraised the study quality using the Downs and Black (modified) checklist. Study outcomes were summarized using a narrative, descriptive method and, where possible, combined for a meta-analysis using a random-effects model.

**Results:**

Of the 301 studies identified in the search strategy, 15 (5%) fair-to-good–quality studies met the inclusion criteria, which identified 10 interventions for JIA (age 4-18.6 years). Of these 10 interventions, 5 (50%) supported symptom monitoring by capturing real-time data using health applications, electronic diaries, or web-based portals to monitor pain or health-related quality of life (HRQoL). Within individual studies, a preference was demonstrated for real-time pain monitoring over recall pain assessments because of a peak-end effect, improved time efficiency (*P*=.002), and meeting children’s and young people’s HRQoL needs (*P*<.001) during pediatric rheumatology consultations. Furthermore, 20% (2/10) of interventions supported physical activity promotion using a web-based program or a wearable activity tracker. The web-based program exhibited a moderate effect, which increased endurance time, physical activity levels, and moderate to vigorous physical activity (standardized mean difference [SMD] 0.60, SD 0.02-1.18; *I*^2^=79%; *P*=.04). The final 30% (3/10) of interventions supported self-management development through web-based programs, or apps, facilitating a small effect, reducing pain intensity (SMD −0.14, 95% CI −0.43 to 0.15; *I*^2^=53%; *P*=.33), and increasing disease knowledge and self-efficacy (SMD 0.30, 95% CI 0.03-0.56; *I*^2^=74%; *P*=.03). These results were not statistically significant. No effect was seen regarding pain interference, HRQoL, anxiety, depression, pain coping, disease activity, functional ability, or treatment adherence.

**Conclusions:**

Evidence that supports the inclusion of eHealth and mHealth interventions in JIA management is increasing. However, this evidence needs to be considered cautiously because of the small sample size, wide CIs, and moderate to high statistical heterogeneity. More rigorous research is needed on the longitudinal effects of real-time monitoring, web-based pediatric rheumatologist–children and young people interactions, the comparison among different self-management programs, and the use of wearable technologies as an objective measurement for monitoring physical activity before any recommendations that inform current practice can be given.

## Introduction

### Background

Juvenile idiopathic arthritis (JIA) is the most common rheumatic disease in pediatric populations [1]. Early diagnosis and active treatment are essential for maintaining physical function and psychological well-being. The treatments aim to control the disease, promote clinical remission, and prevent long-term disability [2-5]. However, to achieve these goals, the management of JIA should be multifactorial [6]. Pediatric-specific issues need tending, such as the use of antirheumatic medications in children and young people, growth retardation, pain and coping, school attendance, psychosocial functioning, dealing with parents, and, in the adolescent years, preparing for the transition to adult care [4,7,8]. For good reason, children and young people need to be closely monitored and supported by specialized rheumatology centers that provide interdisciplinary care using a range of pharmacological, physical, psychological, and educational interventions [6,9-12]. However, several barriers have been identified that hinder this current model of support, delaying the delivery of timely, individualized, and well-coordinated care.

There is an inadequate number of experienced pediatric rheumatologists (PRs) to meet demand and oversee care [7,13-18]. This has resulted in long waiting lists, the centralization of services into tertiary children’s hospitals, and the need for many children and young people to travel long distances to access pediatric rheumatology centers [2,18] or care being delivered by a primary health care provider with no pediatric rheumatology training [2,5,14,18-20]. The World Forum on Rheumatic and Musculoskeletal Diseases clearly states that poor access to health care services can significantly impede diagnosis, appropriate treatment, and health outcomes [14], highlighting the need to overcome these barriers in the delivery of JIA management.

In addition, to achieve optimal health outcomes, children and young people need to adhere to their prescribed treatment plan [21,22], and parents need to support treatment recommendations [2,22]. However, suboptimal rates of adherence are commonly reported [22-24]. For example, a literature review of children and young people with chronic rheumatoid disease reported medication adherence rates as low as 38% and physical activity adherence rates of 40%, particularly during adolescence [22]. The primary reasons included the complexities of chronic disease management and medication schedules, time-consuming nonpharmacological treatments, lack of disease knowledge, and low satisfaction with the health care team [22]. These reasons are not surprising, as a recent systematic review identified 70 studies in which health information was inappropriately tailored to children and young people and their parents’ level of health literacy, increasing their concerns and uncertainties about their condition, treatment options, and shared care decisions [25].

For JIA specifically, further reasons for nonadherence vary across treatment modalities [24] as follows: for oral medications, forgetfulness, taste, and long-term side-effects; for parenteral medications (injectables and infusions), pain and side-effects; and for physical and occupational therapy, forgetfulness, pain, and therapy not being considered necessary [24]. Fortunately, all these reasons are modifiable.

To uphold the expectations of rheumatology care, children and young people should be empowered to take an active role in their disease management by being provided opportunities to improve their health literacy and develop good self-management skills [2,10,25], particularly when considering the long-term benefits these skills will have across their life span. The development of self-management skills is also important as parents only hold a surrogate role in children’s and young people’s health care decisions; therefore, children and young people need to be prepared for their transition from pediatric to adult health care services [26].

A resource-efficient way of supplementing JIA management and the development of self-management skills could be to harness the burgeoning eHealth and mobile health (mHealth) interventions [27]. eHealth is described by the World Health Organization as an activity that delivers health-related information, resources, and services through electronic technology and the internet [28]. mHealth is described as a subbranch of eHealth [28] that uses wireless technology to rapidly uptake, process, and communicate information to support health system efficiency and patient outcomes [29].

The number of studies exploring the potential of eHealth and mHealth interventions for chronic disease management is rapidly increasing. However, most are still at an early stage of development and are limited in their scientific rigor [30-35]; most have been conducted with adults rather than children and young people [31,35], which is interesting, considering that children and young people are experienced users of this form of technology and more likely to use a digital intervention or health app [36-38]. In fact, a recent systematic review identified that children and young people use the internet for at least 1 to 4 hours a day (9438/10,974, 86%) [37] and some type of app every day (719/719, 100%) [39]. Higher rates of use have also been reported for children and young people living with JIA who are at risk of poor psychosocial functioning compared with their peers (>1 hour a day) [36].

However, concerns have been raised about how children and young people use the internet [37,40]. Studies have established that children and young people have poor internet-searching skills, tend to use a 1-word search strategy, briefly skim through search-engine result pages [40], and lack the ability to appraise quality [33,36]. This limits their capacity to find high-quality, personally relevant health information and potentially exposes them to incorrect material [36] or results in them turning to apps and platforms not specifically developed as health resources such as YouTube, Tumblr, and Instagram [41].

Johnson et al [36] believe that for pediatric services to better support the needs of children and young people living with chronic illness, they need to be provided with accessible, developmentally appropriate, and high-quality health-related information. Children and young people with JIA (n=134) agreed, particularly those with low health-related quality of life (HRQoL), expressing an interest in being provided with supportive internet-based interventions [36]. In addition, children and young people participating in feasibility and usability studies and reporting on the delivery of health messages [42], exercise programs [43], symptom monitoring [44,45], and disease management [35,36,44] have also reported high levels of acceptability [42-44], usefulness [35], and satisfaction [43] when using these interventions. However, personal, technical, and device-related barriers have also been identified, which hinder their use [46]. Understandably, before a health care professional can prescribe a digital intervention, it has been suggested that they need at least 3 published papers demonstrating the intervention’s effectiveness [47] to see whether the intervention works in a real-world setting [48].

### Definition of Children and Young People

Internationally, pediatric services cater to children aged 0 to 12 years [49], and adolescents up to the age of 18 years (mean 18.7, SD 2.6 years), before they are transferred to adult services [50]. In this review, we use the term “children and young people” to broadly include all individuals in the age range of 1 to 18 years. We exclude neonates and infants (<1 year) [51].

### Aim and Rationale

This systematic review aims to identify what eHealth and mHealth interventions have proven to be effective in supporting health outcomes for children and young people (aged 1-18 years) living with JIA. We anticipate that this review may aid the clinical use of eHealth and mHealth interventions and their integration in arthritis management.

## Methods

### Overview

This systematic review complies with the PRISMA (Preferred Reporting Items for Systematic Reviews and Meta-Analyses) guidelines [52]. Before commencement of this review, a protocol for this review was registered on PROSPERO (CRD42018108985) [53]. Protocol questions 1, 2, and 4 were presented in a previous study [46], whereas question 3 is presented in this review [53]:

1. What types of eHealth and mHealth interventions have been used to investigate the health care of children and young people diagnosed with JIA?
2. What is the usability of eHealth and mHealth interventions for children and young people diagnosed with JIA?
3. What eHealth and mHealth interventions have proven to be effective in helping children and young people diagnosed with JIA?
4. Are the existing eHealth and mHealth interventions cost-effective for pediatric rheumatology?

### Eligibility Criteria

#### Search Strategy

The search terms in this review were developed by SB after initially searching the National Center for Biotechnology Information Medical Subject Heading terms (Multimedia Appendix 1) [54]. The search terms were adapted to suit 15 health databases with the aim of gaining a broad range of interdisciplinary literature. These databases included MEDLINE or PubMed, Cochrane Library, Joanna Briggs Institute, AMED, CINAHL complete, Embase, JAMA, Informit Health, ProQuest database, PsycINFO, IEEE Xplore, SAGE Publishing, ScienceDirect, Scopus, and Web of Science. The search was conducted in October 2018 and July 2021 and was not restricted by language or year of publication to ensure the inclusion of all relevant studies. Further studies were retrieved from Google Scholar and JMIR and by hand searching reference lists.

Studies retrieved by the search strategy were exported to the web-based platform Covidence [55]. This allowed 2 authors (SB and AC) to independently review titles and abstracts—and then the full-text versions—against the inclusion and exclusion criteria via individual log-ins (Multimedia Appendix 2). The authorship and results of the studies were not masked. Any disagreements that arose were resolved through discussions between SB and AC.

#### Risk of Bias

The Downs and Black [56] (modified) checklist for randomized and nonrandomized studies was used to appraise study quality [57]. Independently, 2 authors (SB and AF) rated 5 main assessment areas—the reporting, external validity, internal validity based on bias, internal validity based on cofounding and selection bias, and power—to provide an overall score out of 28. A score of 24 to 28 was graded excellent, 19 to 23 was graded good, 14 to 18 was graded fair, and <14 was graded poor [57]. Any disagreements between SB and AF in these ratings were resolved through discussion and re-examination of the study.

#### Summary Measures and Synthesis

To assist with data collection, a data spreadsheet was developed using Microsoft Excel to organize the data. Data collection included study characteristics, population, eHealth and mHealth interventions, outcome measurements, and study findings. Data collection was completed by 1 author (SB) and checked by all authors. A narrative synthesis method was used for methodological heterogeneity to identify and present common statistical descriptions [58]. All results were interpreted within the context of each study against the total number of studies and the considered risk of bias.

Where possible, data outcomes from similar studies were pooled, and a meta-analysis was performed to allow the comparison of an intervention group (IG) with a control group (CG). Baseline (time point 1) and end-of-study scores (time point 2) were entered into Review Manager software (RevMan version 5.4) to determine standardized mean differences (SMDs) and 95% CIs [59]. Forest plots were established using continuous data and a random-effects model because of the anticipated effect of clinical heterogeneity and to provide a summary of the distribution of effect [60]. A subanalysis was also conducted to reduce statistical heterogeneity. For the studies examining the same intervention and same fixed parameter, continuous data and a random-effects model were used to consider the common effect of the intervention [61].

Finally, conclusions were drawn by visually inspecting forest plots and interpreting SMDs using the Hedge (adjusted) g. An effect size of 0.2 was considered small, 0.5 was considered medium, and 0.8 was considered large [62]. The presence of heterogeneity was also considered using *I*^2^(*I*^2^=100%×Q [chi-square]−df). A variation of 25% was reported as low, 50% was reported as moderate, and 75% was reported as high [63]. A *P* value of <.05 was considered statistically significant [64].

## Results

### Study Selection

**Figure 1:**
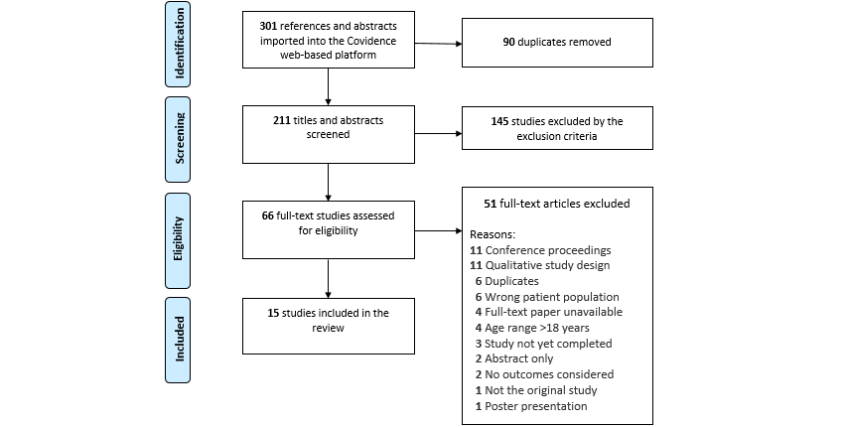
Summary of the study selection process using the PRISMA (Preferred Reporting Items for Systematic Reviews and Meta-Analyses) flow diagram.

The database search retrieved 301 studies. Of the 301 studies, 90 (29.9%) were duplicates; 145 (48.2%) did not meet the inclusion criteria based on their title or abstract; and 51 (16.9%) were excluded in the full-text screening because of study design, population, age range, outcomes, or the inability to gain the full text (eg, abstract only, conference presentations, and posters). Approximately 5% (15/301) of studies met the inclusion criteria to be introduced into this review (Figure 1) [65-79]. Of the 15 studies, only 1 (7%) was retrieved in a language other than English (Dutch), and an English version of the same study was attained through ResearchGate [72].

### Study Characteristics

#### Overview

The 15 studies included in this review were conducted in four countries: Canada [69,70,73-79], the Netherlands [65,67,68,72], the United States [66], and the United Kingdom [71]. These studies were published between 2008 and 2021 (Table 1) [65-79].

**Table 1:** Characteristics of the 15 studies showing population, intervention, control, outcomes, and study design.

First author and country	Population (N) and age range or mean (SD; years)	Intervention	Control (n)	Outcomes	Study design	Dropout (n)
Armbrust et al [65], the Netherlands	49; 8.7-10.8	Rheumates@Work	21^a^	Physical activity (effectiveness)	Multicenter observer blinded RCT^b^	7^c^
Connelly et al [66], United States	289; 12-18	Teens taking charge: managing arthritis on the web	144^d^	Self-management (effectiveness)	2-arm parallel group RCT	24^e^
Doeleman et al [67], the Netherlands	72; 10.6-16.4	EQ-5D-Y-5L^f^ via Ruema2Go App	N/A^g^	HRQoL^h^ monitoring to detect disease activity (efficacy)	Retrospective monocentric study	0^i^
Haverman et al [68], the Netherlands	176; mean 11.6 (SD 4.5)^j^	ePROfile	67	HRQoL (effectiveness) and PR^k^ feedback (n=3)	Sequential cohort study	—^l^
Heale et al [69], Canada	31; 12.8-18.6	Wearable accelerometer using Misfit Flash	N/A	Physical activity (feasibility)	Pre- and postintervention design	3^e^
Lalloo et al [70], Canada	60; mean 15 (SD 1.7)^j^	iCanCope	29^d^	Self-management (feasibility and effectiveness)	2-arm pilot parallel group RCT	12^e^
Lee et al [71], United Kingdom	14; 7-16	My Pain Tracker	1 of 4 rotating groups^d^	Pain (effectiveness)	Randomized N-of-1 crossover trail	0
Lelieveld et al [72], the Netherlands	33; 8-12	Rheumates@Work	16	Physical activity (effectiveness)	Pilot RCT	0
Stinson et al [73], Canada	333; 12-18	Teens taking charge: managing arthritis on the web	169^d^	Self-management (effectiveness)	2-arm parallel group RCT	114^e^
Stinson et al [74], Canada	39; 12-17	iPeer2Peer Program	15^a^	Self-management (feasibility, usability, and effectiveness)	Pilot RCT	9^e^
Stinson et al [75], Canada	70^c^; age not available	eOuch	N/A	Pain (feasibility)	Correlational research	—
Stinson et al [76], Canada	101; 4-18	SUPER-KIDZ	N/A	Pain (efficiency) and PR feedback (n=15)	Descriptive design and 2-stage Delphi technique	—^m^
Stinson et al [77], Canada	46; 12-18	Teens taking charge: managing arthritis on the web	24	Self-management (feasibility)	Pilot RCT	9^c^
Stinson et al [78], Canada	13; 9-18	eOuch	N/A	Pain (feasibility and usability)	Descriptive study	3^n^
Stinson et al [79], Canada	112; 9-17	eOuch	N/A	Pain (feasibility and usability)	Prospective descriptive study	2

^a^Waitlist control.

^b^RCT: randomized controlled trial.

^c^Intention-to-treat analysis.

^d^Active control group.

^e^Excluded in final analysis.

^f^EQ-5D-Y-5L: EuroQol 5-dimensional youth 5-level.

^g^N/A: not applicable.

^h^HRQoL: health-related quality of life.

^i^Data from 4 children and young people were misinterpreted in the assessment and excluded from analysis.

^j^Age range not available.

^k^PR: pediatric rheumatologist.

^l^Not provided.

^m^Pain assessments were completed by parents instead of children (n=4; 4-7 years) and, therefore, excluded from the analysis.

^n^Dropouts (n=3) replaced in phase 2.

#### Participants

A total of 1438 children and young people (range 13-333) were included in this review [65-79]. Studies recruited children and young people from pediatric rheumatology centers or pediatric rheumatology departments within children’s hospitals [65-70,72-79]; one of the studies recruited children and young people from the Childhood Arthritis Prospective Study [71].

Approximately 93% (14/15) of the studies reported on children and young people characteristics [65-74,76-79]. The mean age was 12.97 (SD 1.85) years, varying across studies between 9.7 years and 15.1 years. Most children and young people were female (887/1237, 71.7%) compared with males (350/1237, 28.29%), ranging from 62.9% to 96.7% [65-74,76-79]. The JIA subtypes were aligned with the International League for Rheumatology criteria [80]. Across the study population, the most common subtype of JIA that was reported was oligoarthritis, making up between 21% to 61% [65-74,76-79]. Approximately 7% (1/15) of the studies did not include these characteristics (N=70) [75], and 27% (4/15) of studies excluded the characteristics of children and young people lost during follow-up (n=123) [73,74] or excluded from the final analysis (n=8) [67,76]; (Multimedia Appendix 3 [65-79]).

Approximately 87% (13/15) of the studies considered disease activity [65-70,72-74,76-79]. Reporting either the mean range of disease (range 0.1 to 3.75) using the Physician Global Assessment, Juvenile Arthritis Disease Activity Score or 0-10cm Visual Analogue Scale [65,67-69,74,76-79], or the number of children and young people with low (range 60%-82.5%) scores, or moderate-to-high (range 17.4%-25%) scores [66,70,73], or the number of active (87%, 13/15) and inactive cases (13%, 2/15) [72].

Approximately 20% (3/15) of studies also included feedback from a range of pediatric rheumatology health care providers [67,68,76]. This included PRs (n=18; range 3-15) using eHealth interventions during consultations [68,76] or members of the Childhood Arthritis and Rheumatology Research Alliance (PRs and allied health) replying to a survey (survey 1:115 members; survey 2:157 members [73% replied to survey 1]) or attending a 2-day consensus conference (20 members; pediatric pain and rheumatology experts). Childhood Arthritis and Rheumatology Research Alliance members were from the United States and Canada [76].

#### Interventions

In total, 10 interventions were identified to support children and young people with JIA. The interventions were categorized according to their clinical aim to align with our research question, resulting in the formation of three themes: symptom monitoring, physical activity promotion, and self-management development.

#### Theme 1: Symptom Monitoring

Approximately 33% (5/15) of studies focused on self-reporting pain [71,75,76,78,79]. The interventions used included the following:

1. *My Pain Tracker*, an mHealth app aimed at monitoring pain 1 to 3 times a day or when needed [71]
2. *eOuch*, a customized electronic pain diary aimed at monitoring pain 3 times a day [75,78,79]
3. *SUPER-KIDZ*, a web-based assessment to self-report pain before consultations [76]

Approximately 13% (2/15) of studies focused on self-reporting HRQoL [67,68]. The used interventions included the following:

1. *EuroQol 5-dimensional youth 5-level questionnaire* (EQ-5D-Y-5L), accessed through the Reuma2Go health app aimed at remotely identifying disease activity and the need for treatment adjustments [67]
2. *ePROfile*, a web-based assessment (Kwaliteit van leven in kaart or quality of life map website) aimed at improving HRQoL discussion during rheumatology consultations [68]

#### Theme 2: Physical Activity Promotion

Approximately 20% (3/15) of studies focused on promoting physical activity [65,69,72]. The interventions used included the following:

1. A *wearable activity tracker*—using the commercially available *MisFit Flash*—aimed at improving physical activity levels (PALs) [69]
2. *Rheumates@Work*, a web-based behavioral and cognitive program aimed at delivering health information related to JIA and improving PALs [65,72]

#### Theme 3: Self-management Development

Approximately 33% (5/15) of studies aimed to develop self-management skills [66,70,73,74,77]. The interventions used included the following:

1. *Teens taking charge: managing arthritis online*, which is a web-based behavioral and cognitive program aimed at providing disease-specific information and self-management strategies to improve health outcomes [66,73,77]
2. *iCanCope*, a smartphone app aimed at tracking and improving pain self-management [70]
3. *iPeer2Peer Program*, a web-based peer-mentoring program aimed at facilitating positive role modeling and social support through video calls [74]

The expected level of engagement with the interventions varied from a few minutes before rheumatology consultations to 17 weeks [65-79]. Of the 15 studies, 14 (93%) required the children and young people to use the intervention at home (age range 7-18 years) [65-75,77-79], and only 1 (7%) was conducted in a clinical setting (age range 4-18 years) to monitor use [76]. For a more detailed description of each intervention, see Multimedia Appendix 4 [65-79,81].

#### Comparator or Control

Approximately 53% (8/15) of studies compared a pretested (time point 1) and posttested (time point 2) IG (455/904, 50.3%; range 17-144, median age 12.9, SD 2.09 years; female 322/455, 70.8%) with a CG (449/904, 49.7%; range 14-145, median age 13.4, SD 1.91 years; female 352/449, 78.4%) [65,66,68,70,72-74,77]. Of these 8 studies, 3 (38%) compared the IG with a CG receiving usual care (no eHealth or mHealth input) [68,72,77], 2 (25%) used a waitlist control method to allow all children and young people exposure to the intervention before study completion [65,74], and 3 (38%) compared the IG with an active CG also receiving a digital intervention [66,70,73].

One of the studies compared different real-time reporting schedules across 4 groups (n=12) with a median age of 12.5 years (range 10-14 years; female 9/12, 75%) [71].

#### Outcomes

Study outcomes varied according to the intervention stage of development (feasibility, usability, efficiency, and effectiveness) [65-79]. Health outcomes that considered an evaluation measurement to allow the quantitative comparison between groups, and an effectiveness analysis, were categorized to support the clinical aim of each intervention under the three intervention themes: symptom monitoring, physical activity promotion, and self-management development (Table 2).

**Table 2:** Formation of themes, evaluation criteria, and main outcomes supporting the delivery of the eHealth and mobile health interventions for juvenile idiopathic arthritis.

Theme (interventions aim)			Outcomes (evaluation measurement)
**Theme 1: symptom monitoring**			
	Real-time pain	Pain intensity, unpleasantness, interference using electronic VAS^a^ 5 cm [75,78,79] and RPI^b^ [75,78,79] Pain location and descriptors: size (severity), throb rate (intensity), and emotion, PROMIS^c^ and Pediatric pain Interference Scale–Short Form [71] PedsQL^d^ generic inventory—and arthritis module and PCQ^e^ and Physician Rated Disease Activity Indices [79] Children and young people aged 4-7 years: Faces Pain Scale–Revised; children and young people aged 8-18 years: NRS^f^ (0-10 cm) [76], survey, and consensus conference [76]	
	HRQoL^g^	EuroQol 5-dimensional youth 5-level questionnaire and 0-100 cm VAS (current health status) Juvenile Arthritis Disease Activity Score with 71 joint count [67]	
	Pediatric rheumatology feedback	HRQoL communication during pediatric rheumatology consultation, number of psychologist referrals, and PR^h^ satisfaction [68] Satisfaction questionnaire and 2-stage Delphi survey [76]	
**Theme 2: physical activity promotion**			
	Objective measurements	Bruce Treadmill protocol for exercise capacity (endurance time) [65,72] Accelerometer (Actical Phillips Respironics) for physical activity [65]	
	Self-reporting measurements	A 7-day activity diary [65,72] 3-Day Activity Recall to measure the metabolic equivalent values of activities and PROMIS [69] PedsQoL (version 4) and pain and well-being (0-10 cm VAS); functional ability: CHAQ^i^ [65,69] School absenteeism, participation in physical education classes, and follow-up 3 and 12 months [65]	
	Functional capacity	CHAQ [69] Dutch version CHAQ38 [65]	
	Disease activity	Disease and medication use records [72] Disease activity 0-10 cm VAS [65] Physician Global Assessment 0-10 cm or 0-100 cm VAS [69,72]	
**Theme 3: self-management development**			
	Pain reduction	RPI [74,77] Pain intensity [66,70,73] and interference [66,73] using an 11-point NRS (0-10) [66,70] Tracking logs [70] Follow-up at 3, 6, and 12 months [66,73]	
	HRQoL improvement	PedsQL [66,70,73,74] Juvenile Arthritis Quality of Life Questionnaire [77] PROMIS: pediatric anxiety short form and depressive symptoms short form [66,73] Perceived Stress Questionnaire [77] Follow-up at 3, 6, and 12 months [66,73]	
	Functional capacity	Child Activity Limitations Interview [70]	
	Health literacy	Medical Issues, Exercise, Pain, and Social Support Questionnaire [66,73,74,77] Children’s Arthritis Self-Efficacy scale [66,73,74,77] PCQ (behavioral and cognitive pain-coping strategies) [66,73] Follow-up at 3, 6, and 12 months [66,73]	
	Adherence to prescribed treatment	Child Adherence Report Questionnaire and Parent Adherence Report Questionnaire [73,77]	

^a^VAS: visual analog scale.

^b^RPI: Recall Pain Inventory.

^c^PROMIS: Patient-Reported Outcomes Measurement Information System.

^d^PedsQL: Pediatric Quality of Life Inventory.

^e^PCQ: Pain Coping Questionnaire.

^f^NRS: numeric rating scale.

^g^HRQoL: health-related quality of life.

^h^PR: pediatric rheumatologist.

^i^CHAQ: Childhood Health Assessment Questionnaire.

#### Study Design

Study designs included two 2-arm parallel group randomized controlled trials (RCTs) [66,73], one 2-arm pilot parallel group RCT [70], 1 multisite observer-blinded RCT [65], 3 pilot RCTs [72,74,77], 1 randomized N-of-1 crossover trial [71], 1 descriptive study with 2-stage Delphi technique [76], 1 descriptive study with 2-phase testing [78], 1 prospective descriptive study [79], 1 retrospective monocentric study [67], 1 pre- and postdesign study [69], 1 correlational study [75], and 1 sequential cohort intervention study [68] (Table 1) [65-79].

### Methodological Quality of Studies

Using the Downs and Black [56] (modified) checklist, the overall mean quality score of the 15 studies was 18.87 (SD 1.92) [65-79]. The scores ranged from 15 to 21, providing a fair-to-good score [57] (Multimedia Appendix 5 [65-79]). There were no disagreements between SB and AF that needed to be resolved by a third author (AC). The 2 areas in which study quality was consistently limited were power and sampling bias; 87% (13/15) of studies had insufficient power to detect a clinically significant effect [65,67-78], and convenience sampling and selection bias may have prevented full representation of the JIA population [68,72,75,79]. Children and young people were selected because of pain experience [66,70,78]; level of disease activity [65,66,69,71,75,78,79]; unlikelihood of medication changes [69]; no other comorbidities or cognitive impairments [65,66,69-71,73-75,77,79]; good visual acuity [75,79]; no hand deformities [75,79]; reduced PALs [65]; access to a computer, tablet, or phone or the internet [65,69,70,72,74]; and level of comprehension and ability to speak and read English [65,66,70,73-77,79], Dutch [72], Spanish [66], or French [73,77]. Methodological concerns were also seen in internal validity because of contamination or unreliable compliance [65-67,69-71,73,75-79].

### Results of the Studies

#### Theme 1: Symptom Monitoring

##### Real-time Pain

Approximately 33% (5/15), fair-to-good–quality studies reported on real-time pain assessments [71,75,76,78,79]. Of these 5 studies, 3 (60%) reported on children and young people (aged 11.2-18 years) using *eOuch* to record their pain 3 times a day against the three pain rating measurements: intensity, unpleasantness, and interference [75,78,79], demonstrated a strong correlation (*r*=0.71-0.74, *P*<.01) between these pain measurements [79]. On average, pain scores reported were mild to moderate, interfering mostly with walking and least with school work, relationships with friends or family, and sleeping [78,79]. A good-quality study demonstrated changes in children’s and young people’s pain recordings throughout the day (interference *P*<.01, stiffness *P*<.01, and fatigue *P*<.01) and, week to week (intensity *P*<.01, unpleasantness *P*<.01, interference *P*<.01, and stiffness *P*<.01) [79]. Predicted changes in pain were also seen after a joint injection (medium effect size: 0.52-0.71); the main effect was for pain intensity [79]. A weak effect was reported for tiredness (*r*=0.24-0.26) and perceived ability to control pain (*r*=0.6-0.26) [79]; Multimedia Appendix 6 [71,75,78,79]).

Of the 5 studies, a further 1 (20%) fair-quality study reporting on the intervention *SUPER-KIDZ* that targeted children and young people aged between 4 to 18 years considered the pain dimensions that should be included in a pain assessment [76]. Using a 2-stage Delphi technique, the consensus view from health care experts (survey 1: n=115; survey 2: n=157; 2-day conference: n=20) concluded the inclusion of the characteristics of pain—intensity, location, frequency, duration, and the consequences of pain—and functional limitations [76].

Another 20% (1/5) of fair-quality studies reported on the frequency of recording real-time pain scores using *My Pain Tracker* [71]*.* Children and young people (aged 7-16 years) adherence rates were higher when pain was reported once a week (15/24, 63%) compared with when pain was reported once a day (85/168, 50.6%) or twice a day (127/336, 37.8%) or *as and when* pain was experienced (range 0-7 reports) [71]. There were no significant differences in pain interference scores because of reporting frequency (*P*=.77) or the different time points (weeks) across the study (*r*=−.004; *P*=.68). The children and young people qualitative results reported that they preferred once a day or *as and when* (6/14, 43%) reporting schedules [71] (Multimedia Appendix 6).

##### Real-time Pain Assessments Versus Recall Pain Assessments

Of the 5 real-time pain assessment studies, 3 (60%) fair-to-good–quality studies considered the correlation between *eOuch* real-time pain recordings and the Recall Pain Inventory short form [75,78,79]. For CPY (aged 11.2-18 years), a moderate to strong correlation (*r*=0.49-0.84) was reported between the real-time pain recordings and recall pain recordings (*P*<.01) [79], and the magnitude of changes in pain did not differ significantly when pain was defined as >0/100 or >0/30. However, when pain was defined as >0/10, there was weak *within-person consistency*, resulting in an 8% variance and a moderate association between the 2 assessments [75]. The same study also reported computed changes in pain (*P*=.02) against the judged assessment of pain (*P*=.004), finding both to be significantly similar, although the Recall Pain Inventory was higher and predictable [75]. Recall pain assessment measurements were mostly influenced by the children and young people peak pain score and the last real-time pain score. This finding appeared to be clinically significant (Multimedia Appendix 6).

##### Real-time Pain Scores Versus Other Commonly Used Pediatric Assessments

Of the 5 real-time pain studies, 1 (20%) fair-quality study compared real-time pain scores, using *eOuch*, with other pediatric tools (Pediatric Quality of Life Inventory [PedsQL] Generic Inventory, PedsQL Arthritis Module, and Pain Coping Questionnaire). For children and young people (aged 9-17 years), a weak to moderate correlation (*r*=0.02-0.64) was seen, highlighting differences in the assessment tools, suggesting the need for specific pediatric pain assessments (Multimedia Appendix 6).

##### HRQoL Assessment Versus Disease Activity Assessment

Of the 15 studies, 1 (7%) good-quality study compared children and young people (aged 10.6-16.4 years) self-reporting HRQoL at home, using the *EQ-5D-Y-5L* assessment, with the commonly used clinical care tool Juvenile Arthritis Disease Activity Score with 71 joint count, which was completed by the PR during consultation to measure disease activity [67]. The HRQoL assessment (EQ-5D-Y-5L sum score) across all 5 levels (mobility, self-care, daily activities, pain or discomfort, and anxiety or depression) displayed satisfactory diagnostic accuracy (87%; 95% CI 76-94; *P*<.001), sensitivity (85%), specificity (89%), and predictive values (positive 88% and negative 86%) in identifying moderate to high disease activity [67]. This suggests that disease activity would not have been missed through remote monitoring of HRQoL, and treatment adjustments based on the current-to-treat guidelines (>1.5 for oligoarthritis and >2.5 for polyarthritis) could be applied [67].

##### PR’s Feedback

Of the 15 studies, 1 (7%) good-quality study compared the preferred method of reviewing pain assessments by PRs (11/15, 73% female; 10/15, 67% practicing >10 years) [76]. *SUPERKIDZ* pain assessments were completed by children and young people (aged 4-18 years; with no help from parents) before the PR consultation using three different methods: a laptop or computer, a multimedia player, and a paper-based assessment. PRs (10/15, 67%) reported the electronic assessments to be more time efficient (*P*=.02) than the paper-based assessment and would recommend the use of web-based pain summaries to colleagues (9/15, 60%). There were no differences reported in developing pain management plans (10/15, 67%) [76].

Of the 15 studies, 1 (7%) fair-quality study reported on the PR’s review of the web-based HRQoL assessment, *ePROfile,* during consultation [68]. PRs (n=5) reviewed 176 children and young people (mean 11.6, SD 4.5 years) tabulated answers and were satisfied with the care they provided for the IG compared with the care they provided for the CG, particularly in the areas of emotional support (first consultation [time point 1] *P*<.01 and second consultation [time point 2] *P*<.001) and meeting children and young people needs (time point 1 and time point 2 *P*<.001). PR satisfaction increased slightly in the second consultation compared with that of the first. PR evaluations reported *ePROfile* as useful (time point 1: 97/102, 95%; time point 2: 64/64, 100%), and the number of referrals increased (time point 1=9.2% and time point 2=4.3%) compared with the CG (3%). These results were not significant [68]. Parents also evaluated *ePROfile* as useful (time point 1: 57/65, 88%; time point 2: 37/46, 80%); however, parent satisfaction did not differ between the IG and CG, and children and young people (mean age 11.6, SD 4.5 years) reported the consultation as normal (time point 1: 47/48, 98%; time point 2 29/35, 83%). *ePROfile* was considered by the study authors as an efficient medium for monitoring HRQoL and was implemented in clinical use after the study [68].

#### Theme 2: Physical Activity Promotion

##### Overview

Approximately 20% (3/15) of the fair-to-good–quality studies considered the interventions’ effect on physical activity of children and young people [65,69,72]. Of these 3 studies, 1 (33%) fair-quality study reported on children and young people (aged 12.8-18.6 years) wearing an *activity tracker*, Misfit Flash, daily for 28 days. No significant differences in PALs were recorded [69].

**Figure 2:**
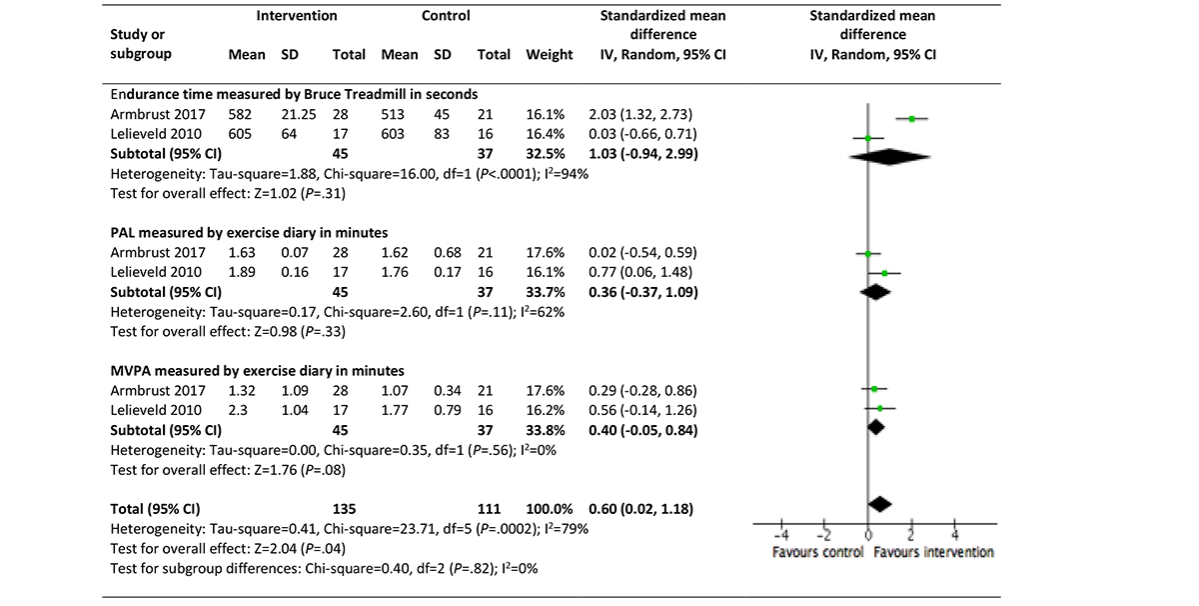
Effectiveness of Rheumates@Work on the promotion of physical activity for juvenile idiopathic arthritis (aged 8-12 years). MVPA: moderate-to-vigorous physical activity; PAL: physical activity level.

The other 67% (2/3) good-quality studies reporting on children and young people (aged 8.7-12 years) who used the intervention *Rheumates@Work* were pooled in a meta-analysis [65,72]. Overall, a moderate effect (SMD 0.60, 95% CI 0.02-1.18, *P*=.40) was seen in physical activity (endurance time, PAL, and moderate to vigorous physical activity [MVPA]). However, there was high statistical heterogeneity between the studies (*I*^2^=79%), suggesting a 79% variance across the studies, reducing confidence in these results (Figure 2) [65,72]. No changes were reported for pain intensity, disease activity, or functional ability [65,69].

##### Seasonal Intervention Effect

Of the 3 studies considering physical activity promotion, 1 (33%) good-quality study, *Rheumates@Work*, reported a seasonal intervention effect after comparing a winter IG to a summer IG. For the winter IG, a 24-minute reduction in rest was recorded using an accelerometer (Actical Phillips Respironics)*.* This result was significant (*P*=.05) [65].

##### Follow-up

Of the 3 studies considering physical activity promotion, only 1 (33%) good-quality study, *Rheumates@Work*, considered follow-up after the study period [65]. At 3 months, for the IG, children’s and young peoples’ (aged 8.7-18 years) physical activity (endurance time and PAL) continued to improve, and by 12 months, it declined. However, this reduction did not reach the preintervention levels. Positive improvements were also reported for educational participation. At 3 months, school absenteeism decreased from 43% to 14% (*P*=.02) in the IG and increased from 24% to 29% (*P*=.60) in the CG. Children’s and young peoples’ participation in physical education classes also improved in the IG group, from 57% to 71% (*P*<.01) and from 62% to 67% in the CG (*P*=.01). However, these differences were not statistically significant [65].

#### Theme 3: Self-management Development

##### Overview

Approximately 33% (5***/***15) of fair-to-good quality studies assessed the health-related benefits of self-management development [66,70,73,74,77].

##### Pain Reduction

Of the 5 studies promoting self-management, all (100%) fair-to-good–quality studies monitored for changes in pain because of the intervention [66,70,73,74,77]. Of these 5 studies, 1 (20%) fair-quality study reported on children and young people (mean age 12, SD 1.7 years) using *iCanCope* [70]. The IG received a pain monitoring and self-management program, and the CG received pain monitoring only. Both groups reported a reduction in pain intensity (IG: 1.73-point reduction; CG: 1.09-point reduction), using a 0 to 10 numerical rating scale. These results were not statistically significant (*P*=.24) [70].

**Figure 3:**
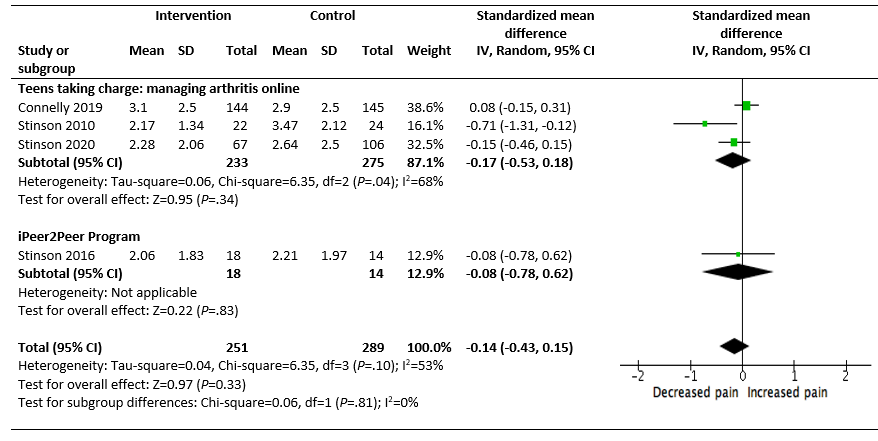
Effectiveness of self-management programs in reducing pain intensity for children and young people (aged 12-18 years) with juvenile idiopathic arthritis.

**Figure 4:**
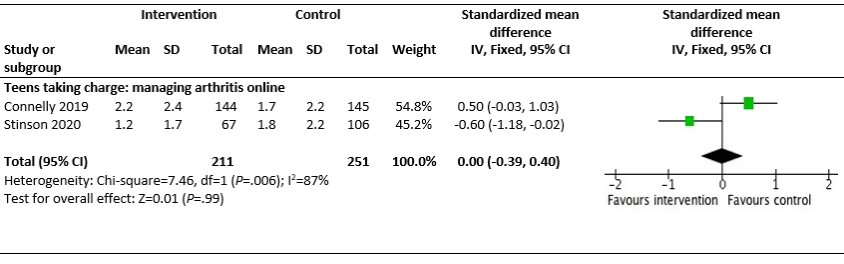
Effectiveness of Teens taking charge intervention in reducing pain interference for children and young people (aged 12-18 years) with juvenile idiopathic arthritis.

Of the 5 studies, 4 (80%) good-quality studies (children and young people aged 8-18 years) reporting on *Teens taking charge* and the *iPeer2Peer Program* were pooled for a meta-analysis [66,73,74,77]. A small postintervention effect was seen in the IG compared with the CG in reducing pain intensity (SMD −0.14, 95% CI −0.43 to 0.15; *I*^2^=53%; *P*=.33). However, these results were not statistically significant, and moderate statistical heterogeneity was seen between the studies (Figure 3) [66,73,74,77]. No effect was seen on pain interference (Figure 4) [66,73].

##### HRQoL Improvements

**Figure 5:**
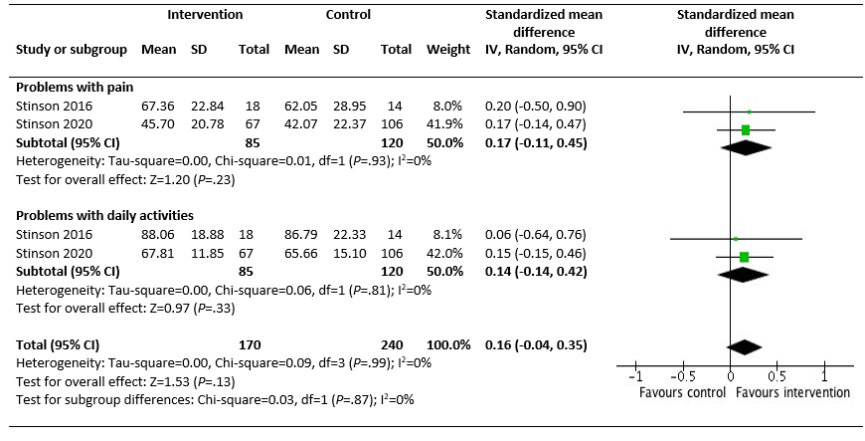
Subanalysis of Teens taking charge intervention and the health-related quality of life domains: problems with pain and daily activities for children and young people (aged 8.7-18 years) with juvenile idiopathic arthritis.

Of the 5 studies targeting self-management development, 4 (80%) fair-to-good–quality studies considered the intervention effect on HRQoL for the IG compared with CG (age range 8.7-18.6 years) [66,70,73,74,77]. Of these 5 studies, 4 (80%) good-quality studies, reporting on *Teens taking charge* and the *iPeer2Peer Program,* were pooled for a meta-analysis. No effect was demonstrated for HRQoL [66,73,74,77]. For *Teens taking charge*, a further subanalysis of the individual HRQoL domains (problems with pain, daily activities, treatment, worry, and communication), using the PedsQL, demonstrated a small effect in improving problems with pain and problems with daily activity (SMD 0.16, 95% CI −0.04 to 0.35; I=0%; *P*=.13) (Figure 5) [73,74]. This effect was not statistically significant. From the study outcomes excluded from the meta-analysis, no improvements were seen in anxiety, depression [66,73], or stress [77].

##### Follow-up

Of the 5 studies targeting self-management development, 2 (40%) good-quality studies considered follow-up after the study period at 3, 6, and 12 months [66,73]. In the Canadian *Teens taking charge* study, children and young people (aged 12-18 years) in the IG retained the improvements they gained during the study period for pain intensity and in the HRQoL domains of problems with pain and problems with daily activities. These results were not statistically significant. A significant improvement was seen in the domain of problems with treatment (*P*=.008) [73]. In the US *Teens taking charge* study, children and young people (aged 12-18 years) in the IG and CG continued to have a stable reduction in pain intensity and pain interference and improvements in HRQoL. The differences between the IG and CG were not significant [66].

##### Health Literacy

**Figure 6:**
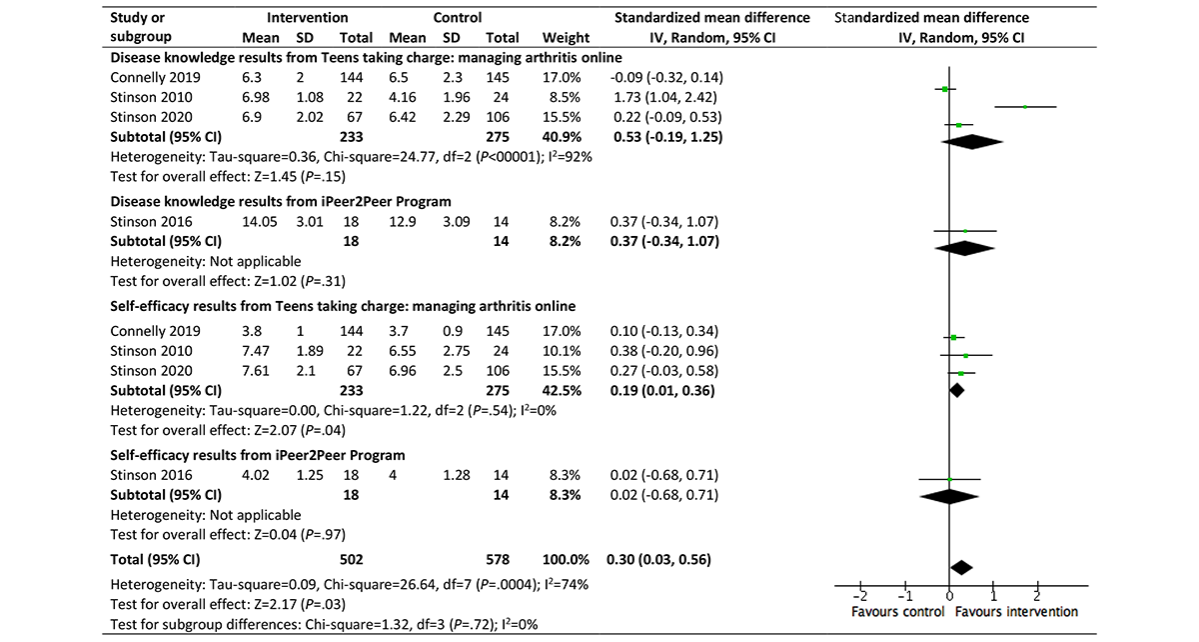
Effectiveness of self-management programs in improving disease knowledge and self-efficacy for children and young people (aged 12-18 years) with juvenile idiopathic arthritis.

Of the 5 studies targeting self-management development, 4 (80%) good-quality studies, reporting on *Teens taking charge* and the *iPeer2Peer Program* (children and young people aged 8.7-18 years) and considering health literacy, were pooled in a meta-analysis [66,73,74,77]. A small, nonsignificant effect was seen in improving disease knowledge and self-efficacy (SMD 0.30, 95% CI 0.03-0.56; I^2^=74%; *P*=.03); however, confidence in these results was reduced because of high statistical heterogeneity (Figure 6) [66,73,74,77]. No improvements were seen in pain coping strategies [66,73].

##### Functional Ability and Adherence to Treatment

Of the 5 studies targeting self-management development, only 1 (20%) fair-quality study reported on functional ability. There was no improvement in pain-related limitations (*P*=.65) [70]. Another 40% (2/5) of good-quality studies reported on treatment adherence. No improvements were reported for medication, exercise, or splint adherence [73,77].

### Adverse Events

Of the 15 studies, 3 (20%) fair-to-good–quality studies recorded adverse events [65,66,69]. *Teens taking charge* (age range 12-18 years) reported the highest number of adverse events (n=72), mostly related to infections (18/72, 25%) and arthritis-related flares (17/72, 24%) [66]. The more serious events involved hospitalization (9/72, 13%) or suicidal thoughts (4/72, 6%). There was no significant difference in adverse events between the IG and CG groups (*P*=.67) [66]. *MisFit Flash* (age range 12.8-18.6 years) also reported illness, injury, or pain (9/28, 32%), including arthritis-related ankle and knee pain (1/28, 4%). However, no significant difference was seen in functionality (mean Childhood Health Assessment Questionnaire score), pain, or active joint count during the study [69]. Whereas *Rheumates@Work* (age range 8-12 years) reported arthritis-related flares, affecting more children and young people in the CG (2/17, 12%) compared with the IG (1/16, 6%) [65].

### Dropout

Of the 15 studies, 10 (67%) studies reported dropout rates (range 0-114) by children and young people (aged 8.7-18.6 years) [65,66,69,70,72-74,77-79]. Dropout reasons before study commencement included not being interested anymore [65,66,74], early withdrawal before allocation [66,77], not receiving allocation [65,70,77], not completing app orientation [70], technical issues [77], and no show and no reason [65]. Reasons during the study period included other health problems [65,69], school and extracurricular activities [69,78], discontinued use [66,74,77], did not complete final web-based measures [77], unable to reach [66], lost to follow-up [66,69,73,74,77,79], and removal because of lack of compliance [74]. No comparisons were made between age or gender [65,66,69,70,72-74,77-79].

Of the 15 studies, 7 (47%) studies reported both the IG and CG dropout rates [65,66,70,72-74,77]. A higher dropout rate was reported in the IG (119/455, 26.2%; range 0-76) compared with the CG (56/449, 12.5%; range 0-56) [65,66,70,72-74,77]. The Canadian *Teens taking charge* study reported the highest dropout rate (IG: 76/164, 46.3%; CG: 38/169, 22.5%) [73].

## Discussion

### Principal Findings

To the best of our knowledge, this is the first systematic review to evaluate the effectiveness of eHealth and mHealth interventions in supporting children and young people living with JIA. In total, 10 interventions were identified to support symptom monitoring, physical activity promotion, or self-management development for children and young people aged 4 to 18.6 years. These 10 interventions included 4 (40%) web-based programs [65,66,68,72,73,76,77], 3 (30%) health applications [67,70,71], 1 (10%) telecommunication application [74], 1 electronic diary (10%) [75,78,79], and 1 (10%) accelerometer compatible with a tablet or smartphone [69]. The methodological quality of the studies supporting these interventions ranged from fair [68-71,75,76,78] to good [65-67,72-74,77,79].

### Theme 1: Symptom Monitoring (4-18 Years)

Pain assessment was the most common type of intervention used to support symptom monitoring. The interventions *My Pain Tracker* and *eOuch* aimed to capture real-time data through children and young people self-reporting pain [71,75,78,79]. Monitoring pain is important as pain is the most frequently reported symptom by children and young people living with JIA [12,82]. Pain can dramatically interfere with physical functioning, coping mechanisms, and quality of life [12]. Stinson et al [79], through the use of *eOuch* pain diaries, demonstrated a correlation between pain intensity and the impact pain can have on emotional well-being (unpleasantness) and activities of daily living (interference), reinforcing the need for ongoing comprehensive pain monitoring, which could allow the health care team to make timely recommendations and prevent poor health outcomes [83-85].

However, there is no consensus on the required number of real-time assessments, per day or week [71,86,87], to ensure the collection of high-quality data and avoid the burden of momentary reporting [71]. Instead, a large variation, ranging from 2 to 9 times a day for children and young people, has been seen [87]. In this review, real-time pain monitoring ranged from 1 to 3 times a day [71,75,78,79] or when needed [71]. Lee et al [71], through *My Pain Tracker*, compared these reporting frequencies, finding that children and young people preferred once-a-week or when-needed pain assessments to avoid thinking about their pain. Although more details in pain data were collected from once-a-day reporting, and, for some children and young people, adherence to once-a-day reporting was easy as it became a routine [71], more research is needed on reporting frequencies.

Real-time pain monitoring also exposed differences between real-time pain and recall pain assessments [75]. Recall pain measurements were higher and predictive compared with average real-time pain measurements, influenced by the children’s and young peoples’ most intense pain and last pain score [75]. This is known as recall bias or peak-end effect [75,88]. This nonequivalence between real-time pain assessments and recall pain assessments adds significance to previous research by Stone et al [88], highlighting methodological concerns around relying on retrospective pain assessments, especially when considering the length between rheumatology appointments.

Longitudinal variances were also seen between real-time and recall pain monitoring [75]. Stinson et al [75] and Stone et al [88] both demonstrated a weak correlation with within-person data when pain was defined as >0/10, which is the most common pediatric pain scale. This suggests that real-time and recall pain assessments cannot be compared or used interchangeably when assessing long-term changes in pediatric pain [75]. Considering that the length of the studies in this review was only 2 to 8 weeks [71,75,78,79], further research on the longitudinal effects of real-time pain monitoring is needed.

The use of real-time symptom monitoring for children and young people is also supported by previous work in reducing the recall time to days, hours, or minutes [87], and importantly, a recent systematic review, reporting on real-time monitoring using mobile technology, suggests that it can be successfully implemented from the age of 7 years [87]. In addition, a study considering adults with chronic illnesses supports real-time monitoring for the identification of exacerbations, confidence in self-management, and prevention of hospital admissions [89]. In this review, the intervention *EQ-5D-Y-5L* endorsed this finding, as remote HRQoL monitoring identified, with satisfactory diagnostic accuracy (*P*<.001), moderate to high levels of disease activity, promoting the need for adjustments with prescribed treatments and rheumatology consultation frequency [67]. Further research is now needed on this web-based PR– children and young people interaction and the impact remote monitoring may have on safety [67,90].

In this review, 13% (2/15) of studies reported positive feedback from PRs after they reviewed web-based assessments during consultation [68,76]. PRs reported that *SUPER-KIDZ* pain assessments increased time efficiency compared with a paper-based assessment [76]. However, *ePROfile* increased PR satisfaction with the care they provided as the HRQoL discussion improved and the number of psychological referrals increased [68]. Although these findings were not significant, reviewing pain and HRQoL during consultation is important as children and young people with JIA have significantly lower HRQoL compared with that of healthy children and young people, and children aged 8 to 12 years with JIA have lower HRQoL than that of children with other chronic health conditions [9].

Interestingly, the use of web-based portals in adult rheumatology has been long standing. The Feed Forward System, for example, used in Sweden generates a patient’s progress over a period and has been successfully used to guide health care provider recommendations and aid the development of patient self-management skills [91].

For JIA, feasibility studies considering web-based portals also support their use, reporting that this form of technology can increase children’s and young peoples’ (aged 5-22 years) feeling of control [92,93].

Regrettably, parents and children and young people did not report the same level of satisfaction with the *ePROfile* consultation as PRs [68]. Haverman et al [68] suggest that this may be as they are already happy with the quality of their care. Nonetheless, many factors that can influence children’s and young peoples’ opinions on digital assessments need to be considered. First, they can be influenced by the assessment experience; they need graphical and tailored feedback to encapsulate their results and catch their interest [94]. In addition, children and young people may not value and understand the importance of monitoring symptoms, disease, and general well-being (mood, fatigue, and functional ability) [44,95] or the need for a person-centered framework that builds partnerships between families and health care teams [92]. Further research on the use of web-based portals for children and young people is needed.

### Theme 2: Physical Activity Promotion (8-18.6 Years)

In this review, 20% (2/10) of interventions, *Rheumates@Work* and the wearable *activity tracker*, Misfit Flash, aimed at improving self-management behavior by promoting physical activity for children and young people (aged 8-18.6 years) [65,69,72]. Of these 2 interventions, only 1 (50%) *Rheumates@Work*, demonstrated a moderate but clinically meaningful effect on physical activity, improving endurance time, PAL, and MVPA for children and young people (aged 8-12 years) [65,72]. This finding is important as children and young people with JIA are less physically active [96,97] and spend more time in sedentary activities than their peers [96]. Improving physical activities helps to retain musculoskeletal function, muscle strength, and functional capacity [98].

In addition, increased physical activity did not exacerbate disease activity or pain in the IG compared with the CG [65,69]. In fact, no significant difference was reported [65,69], and absenteeism from school decreased [65]. These findings are encouraging, especially considering the related impact JIA can have on reducing academic performance, as depicted by Bouaddi et al [99] and Laila et al [100]. Although these findings are limited, they will add to the growing body of evidence reporting that exercise therapy is well-tolerated by children and young people with JIA [98,101,102], further supporting physical activity as a helpful and necessary treatment modality, improving adherence [24,103].

### Theme 3: Self-management Development (8-18 Years)

In this review, 30% (3/10) of interventions—*iCanCope* [70], *Teens taking charge* [66,73,77], and the *iPeer2Peer program* [74]*—*supported self-management development for children and young people (aged 8-18 years). These interventions (including *Rheumates@Work* [65]) are typical behavior change technique interventions used for children and young people [31]. They support self-management through the development of disease-specific knowledge, goal setting, self-management strategies, and social support [66,70,73,74,77].

In this review, identified in the meta-analysis, children and young people participating in the self-management programs *Teens taking charge* and *iPeer2Peer Program* reported a small but nonsignificant improvement in pain intensity, disease knowledge, and self-efficacy scores [66,73,77]. However, high statistical heterogeneity was also seen within the results. This may be because of several reasons. First, a range of comparators was used for the CG. For example, in 2 of the 3 *Teens taking charge* studies, the IG was compared with an active CG rather than usual care. The CG also received an eHealth intervention 12 publicly available health education websites with phone support to support care. Improvements were then seen in both the IG and CG, reducing the mean difference between the groups [66]. Most digital studies primarily focus on a single intervention to demonstrate the intervention effect rather than comparing different digital interventions. However, it is this direct comparison that can reveal a more effective intervention [104]. The CG’s intervention, the use of health care workers signposting quality health education websites to support self-management skills [105,106] and improve well-being [107], is supported in the literature. Therefore, acknowledging the improvements seen in the CG is important, as the use of publicly available websites can be a cost-effective solution for dissipating health information among the masses to support the delivery of health care [28]. For example, a study of adults living with chronic pain (n=20; aged 18-74 years) explained that if they had been provided with quality pain-related information, it might have prevented the desperation and anxiety they experienced, especially during the first few years [107].

Another explanation for the moderate to high statistical heterogeneity may have been that the studies were conducted in different countries, within different health care delivery systems, with different levels of pre-existing support [73]. Differences exist with the pediatric rheumatology workforce worldwide [7,13-18] and within publicly funded and self-funded health care systems [108,109]. Differences also exist in PRs’ opinions on pediatric self-management and the use of an interdisciplinary approach to care [108]. Successful publicly available digital interventions may be a solution to transcending these boundaries and universally improving access to care [28,106]. Further comparisons between different self-management interventions is needed, especially when considering the dropout rates in this review, which, for the self-management programs, were higher in the IG [65,66,70,73,74]. These dropout patterns were similar to a recent systematic review predicting dropout rates in adults, with dropouts occurring at the beginning and over the course of the intervention [110].

There may also be no one-size-fits-all intervention, or there may be a need for a combination of interventions. For example, the *iCanCope* pain self-management application combined 2 interventions (real-time pain monitoring and self-management) and then compared this combination to standalone pain monitoring. This combination demonstrated a greater decrease in pain intensity scores (>1 point, 0-10 on the numerical rating scale) [70]. Although this finding was not significant, the inclusion of self-management programs could be clinically beneficial. Improving and providing effective educational interventions early in childhood should be when children and young people are beginning to develop their health behaviors [23]. Studies have shown that a high level of health literacy can support informed decision-making [111-113]; treatment adherence, especially for nonmedication interventions [113]; and the prevention of chronic health-related problems [22].

Unfortunately, not all the results of this review are promising. Across the studies, the interventions had no effect on pain interference [66,73], HRQoL [65,66,69,70,73,74,77], anxiety, depression, pain coping [66,73], disease activity [69,72], functional ability [65,69,70], or treatment adherence [73,77]. In addition, only 20% (3/15) of studies considered long-term follow-up [65,66,73]. More research is needed to gain wider health-related benefits.

### Limitations

It is essential that several limitations are considered when interpreting the findings of this review. First, our search strategy was restricted to an academic context, using eHealth electronically indexed health databases that publish peer-reviewed journals, rather than apps within commercial stores. This means that our results may not provide a true reflection of the health apps available for JIA. This decision was based on the commonly reported shortcomings of health apps available to the general public that are related to data safety and lack of rigorous testing [114].

Second, the selection criteria in this review deviated from our systematic review protocol [53]. In the protocol, we outlined that the comparator or CG was to receive usual care, with no eHealth or mHealth input. Instead, we included 13% (2/15) of studies comparing an eHealth intervention to another digital intervention [66,73], as a preliminary pilot study of this intervention met our inclusion criteria to be included in this review [77]. This decision enabled us to provide the most up-to-date evidence for this intervention.

Third, our findings in this review supporting the use of real-time monitoring and web-based assessments were based on descriptive summaries. The use of a narrative, descriptive methodology to summarize, synthesize, and report the results is at risk of reporting bias. To reduce this risk, all authors internally reviewed all the stages of this review.

In addition, there were methodological concerns in the data reported by some studies because of performance bias. It was not possible to blind children and young people from the intervention, which could have resulted in a placebo effect. For example, *Rheumates@Work* reported improvements in both the IG and CG for MVPA and participation in physical education classes. Baseline testing may have made the CG more aware of the need to improve their physical activities [65]. The interventions’ true effects may have also been overestimated, as activity levels entered by children and young people in the exercise diaries did not match the accelerometers. Overreporting exercise is not uncommon. Various correlations have been reported between exercise diaries and accelerometer recordings in the general population (*r*=0.52) [115]; among children and adolescents (reliable coefficient ranges *r*=0.5-0.93 and validity coefficient ranges *r*=0.03-0.88), with children being in the lower range [116]; and for JIA (light PAL and MVPA *r*<0.24; rest and PAL *r*=0.41) [117]. Unexpectedly, a 4% inaccuracy has also been identified in accelerometer recordings for JIA in light PALs (effect size 1.2) because of nonwearable periods (aquatic activities and ball games) [117]. Awareness of these possible variations enables correction. For example, Armbrust et al [117] recommend, for research purposes, the use of accelerometer recordings (7-19 days) and an activity diary (>13 days). Another feasible suggestion may be the use of wearable forms of digital technology (ie, a smartwatch) [27]; however, more research is needed to overcome the nonwearable periods such as contact sports [118] or while attending school [119].

Finally, the generalizability of our findings may be limited. We included 40% (6/15) of studies where several children and young people were categorized as unknown, not yet diagnosed, or other (55/1438, 3.82%; range 2-37) [68,70,73,76,77,79]. Dissecting the results to target children and young people specifically with JIA was not possible (Table 2). For a fair-quality study, which reported the highest number of children and young people in this category (37/101, 36.6%), our data extraction focused on the consensus view of pediatric rheumatology providers (PRs, allied health experts, and pain experts) rather than the children and young people [76], which is an area of research that is currently limited.

### Conclusions

Evidence that supports the inclusion of eHealth and mHealth interventions in JIA management is on the rise; however, this evidence needs to be considered cautiously. Confidence in the results is reduced because of low sample size, wide CIs, high statistical heterogeneity, and no similar effect being seen across similar studies. More rigorous research is needed that focuses on the longitudinal effects of real-time monitoring, web-based PR–children and young people interactions, comparison of self-management strategies, and the use of wearable digital technology as an objective measurement for monitoring physical activity before any recommendations informing current practice can be given.

## Appendix

Multimedia Appendix 1 Search terms and search strategy.

Multimedia Appendix 2 Inclusion and exclusion criteria.

Multimedia Appendix 3 Juvenile idiopathic arthritis subtypes based on the International League for Rheumatology criteria.

Multimedia Appendix 4 Overview of the eHealth and mobile health interventions used for juvenile idiopathic arthritis.

Multimedia Appendix 5 Methodological scores of the 15 studies using the Downs and Black [56] (modified) checklist.

Multimedia Appendix 6 Real-time pain monitoring versus other tools commonly used in pediatric rheumatology or pediatrics.

## Abbreviations

CG: control group
EQ-5D-Y-5L: EuroQol 5-dimensional youth 5-level
HRQoL: health-related quality of life
IG: intervention group
JIA: juvenile idiopathic arthritis
mHealth: mobile health
MVPA: moderate to vigorous physical activity
PAL: physical activity level
PedsQL: Pediatric Quality of Life Inventory
PR: pediatric rheumatologist
PRISMA: Preferred Reporting Items for Systematic Reviews and Meta-Analyses
RCT: randomized controlled trial
SMD: standardized mean difference
