# Supplementary material for: Effectiveness of eHealth and mHealth Interventions Supporting Children and Young People Living With Juvenile Idiopathic Arthritis: Systematic Review and Meta-analysis
Source: J Med Internet Res. 2022 Feb 2;24(2):e30457. doi: 10.2196/30457 (PMC8851322; doi:10.2196/30457)
Supplement: Multimedia Appendix 1 [file jmir_v24i2e30457_app1.docx]

## **Search terms**

1. eHealth or mobile health OR mhealth OR telehealth
2. child* OR adolescen* or teen* OR youth* OR young pe*
3. juvenile idiopathic arthritis OR juvenile chronic arthritis OR juvenile arthritis

## **Search strategy for the electronic database Medline**

((eHealth or mobile health or mhealth or telehealth) AND (child* or adolescen* or teen* or youth* or young pe*) AND (juvenile idiopathic arthritis or juvenile chronic arthritis or juvenile arthritis)).af.

Data base: Ovid MEDLINE(R) and Epub Ahead of Print, In-Process, In-Data-Review & Other Non-Indexed Citations and Daily <1946 to November 09, 2020>

| **#** | **Query** | **Results from 11 Nov 2020** |
| --- | --- | --- |
| 1 | ((eHealth or mobile health or mhealth or telehealth) and (child* or adolescen* or teen* or youth* or young pe*) and (juvenile idiopathic arthritis or juvenile chronic arthritis or juvenile arthritis)).af. | 4 |
