# Supplementary material for: Effectiveness of eHealth and mHealth Interventions Supporting Children and Young People Living With Juvenile Idiopathic Arthritis: Systematic Review and Meta-analysis
Source: J Med Internet Res. 2022 Feb 2;24(2):e30457. doi: 10.2196/30457 (PMC8851322; doi:10.2196/30457)
Supplement: Multimedia Appendix 2 [file jmir_v24i2e30457_app2.docx]

Table 1: Inclusion criteria

| Criteria | Description of inclusion criteria |
| --- | --- |
|  |  |
| Population | All CYP (1-18 years) diagnosed with JIA using the ILAR (International League of Associations for Rheumatology) criteria |
| Intervention | Any eHealth or mHealth interventions (see definition in the introduction) |
| Control | Any comparator or control group: including usual care (standard face-to-face consultation), wait list control group, active control groups (also receiving an eHealth or mHealth intervention) |
| Outcomes | Any health-related outcome related to the use of eHealth and mHealth |
| Study Design^a^ | Descriptive studies, randomized controlled trials (RCTs), quasi-RCTs, longitudinal and cohort studies |

1. Exclusion: Studies not reporting outcome measures; or reviews, commentaries, or qualitative studies
