# Supplementary material for: Effectiveness of eHealth and mHealth Interventions Supporting Children and Young People Living With Juvenile Idiopathic Arthritis: Systematic Review and Meta-analysis
Source: J Med Internet Res. 2022 Feb 2;24(2):e30457. doi: 10.2196/30457 (PMC8851322; doi:10.2196/30457)
Supplement: Multimedia Appendix 3 [file jmir_v24i2e30457_app3.docx]

### JIA subtypes based on the International League of Associations for Rheumatology [ILAR] criteria

| First author,  JIA subtypes (n) | Polyarthritis (RF-) | Polyarthritis (RF +) | Oligoarthritis | Enthesitis-related | Psoriatic | Systemic | Undifferentiated | Unknown/not yet diagnosed/other | Not recorded c |
| --- | --- | --- | --- | --- | --- | --- | --- | --- | --- |
|  |  |  |  |  |  |  |  |  |  |
| Armbrust [65] | 18 | 2 | 19 | 2 | 2 | 6 | - | - | - |
| Connelly [66] | 130^a^ | - | 60 | 99^b^ | ^b^ | ^b^ | ^b^ | ^-^ | - |
| Doeleman [67] | 15 | 5 | 33 | 5 | 7 | 1 | 2 | - | 4^c^ |
| Haverman [68] | 72 | 7 | 63 | 17 | - | 8 | 7 | 2 | - |
| Heale [69] | 4 | 11 | 7 | 2 | 4 | 3 | - | - | - |
| Lalloo [70] | 14 | 3 | 14 | 12 | 7 | 3 | 3 | 2 | 2^d^ |
| Lee [71] | 5 | 1 | 7 | - | 1 | - | - | - | ^_^ |
| Lelieveld [72] | 9 | - | 20 | - | - | 4 | - | - | - |
| Stinson [73] | 50 | 19 | 71 | 35 | 23 | 5 | 9 | 7 | 114^d^ |
| Stinson [74]^e^ | 7 | 6 | 10 | 1 | 8 | - | - | - | 9^d^ |
| Stinson [75] | - | - | - | - | - | - | - | - | 70 |
| Stinson [76] | 16 | 5 | 9 | 10 | 4 | 6 | 6 | 37 | 8^d,f^ |
| Stinson [77] | 11 | 3 | 14 | 9 | 3 | 3 | - | 3 | - |
| Stinson [78] | 8 | - | 4 | - | 1 | - | - | - | - |
| Stinson [79] | 41 | 6 | 25 | 12 | 10 | 14 | - | 4 | - |

Abbreviation: Rheumatic factor (RF),

1. Including RF-, RF+ and RF unknown
2. Including enthesitis-related, psoriatic, systemic, and undifferentiated
3. Not recorded due to errors in the assessment by CYP [67]
4. Missing data by study author, due to maths error [70], due to drop-out [73,74]
5. CYP (n=2) included twice in subtype groups, due to wait list control. [74]
6. CYP excluded (n=4) because parents completed pain assessment [76]
