# Supplementary material for: Effectiveness of eHealth and mHealth Interventions Supporting Children and Young People Living With Juvenile Idiopathic Arthritis: Systematic Review and Meta-analysis
Source: J Med Internet Res. 2022 Feb 2;24(2):e30457. doi: 10.2196/30457 (PMC8851322; doi:10.2196/30457)
Supplement: Multimedia Appendix 4 [file jmir_v24i2e30457_app4.docx]

**Overview of the eHealth and mHealth interventions used for JIA**

| Theme, name of intervention | | | |
| --- | --- | --- | --- |
|  | | | |
| Theme 1: Symptom Monitoring | | | |
|  | *My Pain Tracker* – real-time pain reporting [71] | | |
|  |  | Program | iPad application (version 1.6.5), multidimensional remote pain monitoring |
|  |  | Intervention classification^a^ | 1.4 Personal Health Tracking,  1.4.3 Active capture/ documentation by client |
|  |  | Additional support | - |
|  |  | Setting, level of engagement | Home, 5 minutes, once-a-day, twice-a-day, once-a-week, and when pain is experienced, for 8 weeks |
|  | *eOuch –* real-time pain reporting [75,78,79] | | |
|  |  | Program | Hand held Personal Digital Assistant (Tungsten W), custom designed software (Grand Pal), multidimensional remote pain monitoring |
|  |  | Intervention classification^a^ | 1.1 Target communication to clients,  1.1.3 Transmit targeted reminders to client(s),  1.4 Personal Health Tracking,  1.4.3 Active capture/ documentation by client |
|  |  | Additional support | 24 hr telephone support for technical problems (via a pager), an audible alarm reminds participants to self-report pain |
|  |  | Setting, level of engagement | Home, three times a day for 2 to 3 weeks |
|  | *SUPER-KIDZ -* web-based pain assessment [76] | | |
|  |  | Program | SUPERKIDZ website, to provide a concise pain summary |
|  |  | Intervention classification^a^ | 1.4 Personal Health Tracking,  1.4.3 Active capture/ documentation by client,  2.3 Healthcare provider decision support,  2.3.3 Screen clients by risk or other health status |
|  |  | Additional support | CYP completed the assessment without help from parents |
|  |  | Setting, level of engagement | Clinic, before consultation |
|  | *EuroQol five-dimensional youth questionnaire* (EQ-5D-Y-5L) – remote monitoring of disease activity [67] | | |
|  |  | Program | Delivered via the Reuma2Go application, targets 5 HRQoL domains using a 5-level classification system; and 0-100cm VAS to rate current health status |
|  |  | Intervention classification^a^ | 2.3 Healthcare provider decision support,  2.3.3 Screen clients by risk or other health status |
|  |  | Additional support | - |
|  |  | Setting, level of engagement | Home, before consultation |
|  | *ePROfile* - web-based HRQoL questionnaire [68] | | |
|  |  | Program | KLIK Website, concerning answers tabulated |
|  |  | Intervention classification^a^ | 2.3 Healthcare provider decision support,  2.3.3 Screen clients by risk or other health status |
|  |  | Additional support | Questionnaire retrieved by PR during consultation |
|  |  | Setting, level of engagement | Home, before consultation |
| Theme 2: Physical activity promotion | | | |
|  | *Wearable activity tracker – accelerometer* [69] | | |
|  |  | Program | Misfit Flash, commercially available fitness tracker, water resistant (30m) |
|  |  | Intervention classification^a^ | 1.4 Personal Health Tracking,  1.4.3 Active capture/ documentation by client |
|  |  | Additional support | Synchronised to compatible smart phone for feedback on activity and daily goals |
|  |  | Setting, level of engagement | Home, 24 hours a day for 28 days |
|  | *Rheumates@Work* ***-***  - web-based program [65,72] | | |
|  |  | Program | Web-based, cognitive behavioural program based on Pender health promotion model |
|  |  | Intervention classification^a^ | 1. Targeted Client Communication,  1.1.2 transmit targeted health information to clients(s) based on health status or demographics |
|  |  | Additional support | 4 group sessions |
|  |  | Level of engagement | Home, 1 hour a week for 14 weeks or 17 weeks [65,72] |
| Theme 3: Self-management development | | | |
|  | *Teens Taking Charge*: *Managing Arthritis Online* - self-management program [66,73,77] | | |
|  |  | Device, program | Web-based, cognitive behaviour program |
|  |  | Intervention classification | 1 Targeted Client Communication,  1.1.2 transmit targeted health information to clients(s) based on health status or demographics |
|  |  | Additional support | Weekly telephone support by trained coach, discussion board |
|  |  | Setting, level of engagement | Home, 20-30 minutes a week, for 12 weeks |
|  | *iCanCope* pain self-management application [70] | | |
|  |  | Program | Mobile application, cognitive behavioural program , evidenced based, targeting CYP with persistent pain |
|  |  | Intervention classification^a^ | 1. Targeted Client Communication,  1.1.2 transmit targeted health information to clients(s) based on health status or demographics  1.4 Personal Health Tracking,  1.4.3 Active capture/ documentation by client |
|  |  | Additional support | Compatible smartphone (iOS/Android), app orientation |
|  |  | Level of engagement | 8 weeks |
|  | *iPeer2Peer Program* - communication application, [74] | | |
|  |  | Program | Computer compatible with Skype^TM^ software, using positive role modelling and social support to improve self-management |
|  |  | Intervention classification^a^ | 1. Targeted Client Communication,  1.1.2 transmit targeted health information to clients(s) 1.3 Client to client communication,  1.3.1 Peer groups for client |
|  |  | Additional support | Discussion board, chat room |
|  |  | Level of engagement | 10 sessions (20-30 minutes) for 8 weeks |

1. World Health Organisation. Classification of Digital Health Interventions: A shared langauge to describe the uses of digistal technology for health. 2018:1-20. URL: <https://apps.who.int/iris/handle/10665/260480> [assessed 2021-07-10]
