# Supplementary material for: Effectiveness of eHealth and mHealth Interventions Supporting Children and Young People Living With Juvenile Idiopathic Arthritis: Systematic Review and Meta-analysis
Source: J Med Internet Res. 2022 Feb 2;24(2):e30457. doi: 10.2196/30457 (PMC8851322; doi:10.2196/30457)
Supplement: Multimedia Appendix 5 [file jmir_v24i2e30457_app5.docx]

**Methodological scores of the 15 studies included in this review, using the Down and Black (modified) checklist**

| First author, year | Reporting | External Validity | Internal Validity  (bias) | Internal Validity  (cofounding, selection bias) | Power | Total score | Judgement |
| --- | --- | --- | --- | --- | --- | --- | --- |
| Score  Range | 0-11 | 0-3 | 0-7 | 0-6 | 0-1 | 28^a^ |  |
|  |  |  |  |  |  |  |  |
| Armbrust, 2017 [65] | 10 | 1 | 4 | 6 | 0 | 21 | Good |
| Connelly, 2019 [66] | 10 | 2 | 4 | 4 | 1 | 21 | Good |
| Doeleman, 2021 [67] | 9 | 3 | 4 | 4 | 0 | 20 | Good |
| Haverman, 2013 [68] | 8 | 3 | 5 | 2 | 0 | 18 | Fair |
| Heale, 2018 [69] | 9 | 1 | 3 | 4 | 0 | 17 | Fair |
| Lalloo, 2012 [70] | 8 | 1 | 4 | 4 | 0 | 17 | Fair |
| Lee, 2020 [71] | 8 | 2 | 4 | 4 | 0 | 18 | Fair |
| Lelieveld, 2010 [72] | 11 | 2 | 5 | 3 | 0 | 21 | Good |
| Stinson, 2020 [73] | 10 | 2 | 4 | 5 | 0 | 21 | Good |
| Stinson, 2016 [74] | 9 | 1 | 5 | 4 | 0 | 19 | Good |
| Stinson, 2014 [75] | 8 | 1 | 4 | 2 | 0 | 15 | Fair |
| Stinson, 2012 [76] | 8 | 2 | 4 | 4 | 0 | 18 | Fair |
| Stinson, 2010 [77] | 8 | 3 | 4 | 6 | 0 | 21 | Good |
| Stinson, 2008 [78] | 8 | 1 | 4 | 4 | 0 | 17 | Fair |
| Stinson, 2008 [79] | 9 | 2 | 4 | 3 | 1 | 19 | Good |

1. Total scores: Excellent: 24-28, good: 19-23, fair: 14-18, poor: less than 14.
