# Supplementary material for: Effectiveness of eHealth and mHealth Interventions Supporting Children and Young People Living With Juvenile Idiopathic Arthritis: Systematic Review and Meta-analysis
Source: J Med Internet Res. 2022 Feb 2;24(2):e30457. doi: 10.2196/30457 (PMC8851322; doi:10.2196/30457)
Supplement: Multimedia Appendix 6 [file jmir_v24i2e30457_app6.docx]

|  | Pain ratings | Correlation | *P* value | Significance of results |
| --- | --- | --- | --- | --- |
|  |  |  |  |  |
| eOuch – correlation between pain ratings [79] | | |  |  |
|  | Intensity, unpleasantness | r=0.73^a^ | *P*<.01 | Strong relationship between the 2 pain ratings |
|  | Intensity, interference | r=0.71^a^ | *P*<.01 | Strong relationship between the 2 pain ratings |
|  | Unpleasantness, interference | r=0.74^a^ | *P*<.01 | Strong relationship between the 2 pain ratings |
| eOuch – detecting changes in pain, 3 weeks after a joint injection [79] | | | |  |
|  | Intensity, unpleasantness, interference | r=0.71, r=64, r=52^a^ | *P*<.01 *P*<.01 *P*<.01 | Construct validity – moderate to strong |
|  | Stiffness | r=0.60 | *P*<.01 | Moderate |
|  | Tiredness | r=0.26 |  | Weak |
|  | Perceived ability to control pain | r=0.26 |  | Weak |
| eOuch vs Recall Pain Inventory [75,79] | |  |  |  |
|  | Intensity, unpleasantness, interference [79] | r=0.49 to 0.84^a^ | *P*<.01 | Moderate to strong relationship between the 2 tools |
|  | Between-person consistency^a,b^ [75] | Week 1: r=0.55 Week 2: r=0.76^a^ Week 1: r=0.53, Week 2: r=0.76^b^ |  | Moderate to strong consistency^a^, and similar patterns^b^, between the 2 tools. |
|  | Between-person agreement [75] | Week 1: r=0.52, Week 2: r=0.75^c^ |  | Similar observed differences in pain in both tools |
|  | Within-person consistency and agreement >0/100 or >30/100 pain scale [75] | Pain >0/100: r=0.29^a^ r=0.26^b^  r=0.25^c^ Pain >30/100: r=0.30^a^ r=0.29^b^ r=0.29^c^ | *P*=.6 | Changes in pain did not differ significantly |
|  | Within-person consistency: 0>10 pain scale [75] | 8% variance between the tools |  | Changes in pain differed between the two tools. Tools not interchangeable when assessing changes in pain over time. |
|  | Within-person agreement [75] | Pain >0/100: r=0.25, Pain >0/30: r=0.29^c^ |  | Similar consistency |
|  | Last reported pain [75] | Week 1: r=0.42,  Week 2: r=0.68^a^ | *P*=.0002 *P*<.0001 | Peak end effect influenced recall pain |
|  | Peak (maximum) pain [75] | Week 1: r=0.50  Week 2: r=75^a^ | *P*<.0001 *P*<.0001 | Peak end effect influenced recall pain |
| eOuch vs other tools [79] | |  |  |  |
|  | PedsQL Generic Inventory  PedsQL Arthritis Module | HRQOL: r=-0.39 to -0.64^a^  Disease-specific HRQL: r=-0.18 to -0.58^a^ |  | Discriminant validity:  tools not related |
|  | Pain Coping Questionnaire | Emotion-focused pain coping: r=0.24 to 0.48^a^ |  | Tools not related |
|  | Physician related disease activity | r=0.02 to 0.27^a^ |  | Tools not related |
| My pain tracker vs Patient-Reported Outcome Measurements Information System (PROMIS) Pain Interference Short Form (8-16 years); or Proxy Pediatric Pain Interference Scale Short-form [71] | | | | |
|  | Pain interference scores, week to week | r=-0.04^b^ | *P*=.68 | Reliability of pain scores, no difference |
|  | Pain interference and pain reporting frequency (1 a day, 2 a day, once a week, or when pain is experienced) | twice a day r=0.73, one a day r=0.93, once a week r=0.63, when pain is experienced r=0.90^b^ | *P*=.77 | No difference |
|  | Once a day pain reporting frequency vs other frequency rates | twice a day, once a week, or when pain is experienced^b^ | *P*=.59 *P*=.98 *P*=.56 | No difference |

a. Pearson interclass correlation

b. Intraclass correlation (ICC C1)

c. Intraclass correlation (ICC, A1)
